# Effects Due to Rhizospheric Soil Application of an Antagonistic Bacterial Endophyte on Native Bacterial Community and Its Survival in Soil: A Case Study with *Pseudomonas aeruginosa* from Banana

**DOI:** 10.3389/fmicb.2016.00493

**Published:** 2016-04-26

**Authors:** Pious Thomas, Aparna C. Sekhar

**Affiliations:** Endophytic and Molecular Microbiology Laboratory, Division of Biotechnology, ICAR-Indian Institute of Horticultural ResearchBengaluru, India

**Keywords:** antagonistic effect, bacterial endophytes, banana, biological control, microbe–microbe interactions, *Musa* sp., *Pseudomonas aeruginosa*, soil microbial community

## Abstract

Effective translation of research findings from laboratory to agricultural fields is essential for the success of biocontrol or growth promotion trials employing beneficial microorganisms. The rhizosphere is to be viewed holistically as a dynamic ecological niche comprising of diverse microorganisms including competitors and noxious antagonists to the bio-inoculant. This study was undertaken to assess the effects due to the soil application of an endophytic bacterium with multiple pathogen antagonistic potential on native bacterial community and its sustenance in agricultural soil. *Pseudomonas aeruginosa* was employed as a model system considering its frequent isolation as an endophyte, wide antagonistic effects reported against different phytopathogens and soil pests, and that the species is a known human pathogen which makes its usage in agriculture precarious. Employing the strain ‘GNS.13.2a’ from banana, its survival in field soil and the effects upon soil inoculation were investigated by monitoring total culturable bacterial fraction as the representative indicator of soil microbial community. Serial dilution plating of uninoculated control versus *P. aeruginosa* inoculated soil from banana rhizosphere indicated a significant reduction in native bacterial cfu soon after inoculation compared with control soil as assessed on cetrimide- nalidixic acid selective medium against nutrient agar. Sampling on day-4 showed a significant reduction in *P. aeruginosa* cfu in inoculated soil and a continuous dip thereafter registering >99% reduction within 1 week while the native bacterial population resurged with cfu restoration on par with control. This was validated in contained trials with banana plants. Conversely, *P. aeruginosa* showed static cfu or proliferation in axenic-soil. Lateral introduction of soil microbiome in *P. aeruginosa* established soil under axenic conditions or its co-incubation with soil microbiota in suspension indicated significant adverse effects by native microbial community. Direct agar-plate challenge assays with individual environmental bacterial isolates displayed varying interactive or antagonistic effects. In effect, the application of *P. aeruginosa* in rhizospheric soil did not serve any net benefit in terms of sustained survival. Conversely, it caused a disturbance to the native soil bacterial community. The findings highlight the need for monitoring the bio-inoculant(s) in field-soil and assessing the interactive effects with native microbial community before commercial recommendation. varying interactive or antagonistic effects. In effect, the application of *P. aeruginosa* in rhizospheric soil did not serve any net benefit in terms of sustained survival. Conversely, it caused a disturbance to the native soil bacterial community. The findings highlight the need for monitoring the bio-inoculant(s) in field-soil and assessing the interactive effects with native microbial community before commercial recommendation.

## Introduction

With increasing awareness about the hazardous effects of agrochemicals employed in crop husbandry, there is an impetus on the usage of safe and effective microorganisms in agriculture toward protection against biotic and abiotic stresses and in crop production (Zarb et al., 2005; Thomas and Upreti, 2015). It is often observed that the growth promotion effects or the antagonistic potential shown by the bacterial strains in laboratory assays are not translated to effective biocontrol strategies in the field. The conditions in the field are different influenced by soil, water, and edaphic factors as well as the native microbial community (van Veen et al., 1997; Bakker et al., 2013; Tyc et al., 2014). The successful performance of a bio-inoculant in the agricultural field is governed by its ability to survive in field soil and the interactive effects with the native microbiome. The introduced organisms in soil are vulnerable to physical stresses and antagonistic effects by other microorganisms (Acea et al., 1988; van Veen et al., 1997). It is also important that the candidate bio-inoculant shall not cause undue biological perturbation in the native soil microbial community. Effects due to the introduced organisms on resident soil microcosm is a topic of much interest to the microbiologists (Trabelsi and Mhamdi, 2013; Tyc et al., 2014).

A gradual reduction in the population of the introduced organism in soil/field has been documented in several instances (van Veen et al., 1997; Matos et al., 2005; Kröber et al., 2014). As for the interaction effects, studies employing *Azospirillum brasilense* in maize rhizospehere (Herschkovitz et al., 2005) or *Bacillus amyloliquefaciens* in lettuce rhizosphere through molecular tools (Chowdhury et al., 2013; Kröber et al., 2014) have indicated only little or marginal changes in the rhizosphere bacterial community. The bioinoculant consortium of *Bacillus megaterium*, *Pseudomonas fluorescens*, and *Trichoderma harzianum* also did not impart any negative effects on rhizospheric microbial community (Gupta R. et al., 2015). On the other hand, significant modifying effects due to the introduced biocontrol agent *Pseudomonas jessenii* on the soil bacterial community composition of lettuce rhizosphere influenced by soil type and time span after inoculation have been documented (Schreiter et al., 2014). The microbial community varies from soil to soil and location to location. This is largely constituted by non-cultivable organisms whose community profiles can be studied deploying molecular tools (Ogram et al., 2007; Bakker et al., 2013). Cultivation based methods may not provide a full account of metabolically active cells, yet it can be a powerful tool in different spheres of microbiology and in the modern era of genomics towards exploitation of beneficial organisms or for further physiological, molecular and application studies (Steward and Rappe, 2007; Epstein, 2013; Prakash et al., 2013). Here, we consider that monitoring the cultivable bacterial community in a confined environment could serve as a representative of soil microbiota to assess the effects due the introduced organism.

Endophytic microorganisms are plant internal inhabitants and are often isolated from surface sterilized tissue or through vacuum extraction (Hallmann et al., 1997; Hardoim et al., 2015). Endophytes are known to benefit plants through growth promotion and antagonistic effects on phytopathogens and pests besides facilitating phytoremediation (Ryan et al., 2008; Gaiero et al., 2013; Kumar et al., 2014). The potential for intra-tissue colonization and their intimate association with the host (Thomas and Reddy, 2013; Thomas and Sekhar, 2014) make endophytes more valuable agents in biocontrol applications over the rhizospheric organisms (Turner et al., 2013; Upreti and Thomas, 2015). Endophytes are normally considered to be recruited by the host plant primarily from the soil community through roots (Hallmann et al., 1997; Hardoim et al., 2015). The methods for the delivery of endophytes in agriculture range from inoculation of seed, seedling or other planting propagules to soil drenching, stem injection, and foliar sprays (Hallmann et al., 1997; Puri et al., 2015). When an antagonistic microorganism is to be employed as a biocontrol agent against soil-borne pathogens or pests, it warrants that the organism be applied through soil-drenching to neutralize the pathogen/pest in the root-zone from where the endophytes should migrate to the host. In this respect, the survival of endophytic microorganisms under field conditions for a longer duration assumes significance. Endophytes invariably return to the soil at the end of the life span of the host/organ and are thus having a phase in the soil environment before re-colonizing the host (Thomas and Soly, 2009).

Recently, we cultured several bacterial endophytes from the sucker-derived shoot-tips of banana (*Musa* sp.) cv. Grand Naine (Sekhar and Thomas, 2015). The prime target was to explore by testing these isolates for potential biocontrol of banana wilt disease caused by the soil-borne vascular pathogen *Fusarium oxysporum* f. sp. *cubense* (*Foc*), a serious limiting factor in major banana growing areas world over (Stover, 1962; Ploetz et al., 2003). Evaluating the 47 endophytic banana strains against *Foc*, one isolate (GNS.13.2a) exhibited significant antagonistic activity against the pathogen in direct *in vitro* challenge assays and this isolate was identified as *Pseudomonas aeruginosa* (Sekhar and Thomas, 2015). This isolate also displayed antagonistic effects against the soil-borne pathogens, *Ralstonia solanacearum* from tomato and *Erwinia carotovora* from banana and thus a potential biocontrol agent against multiple diseases. *P. aeruginosa* is also known to be a human pathogen and there are concerns about its usage in agriculture (Matos et al., 2005; Kumar et al., 2013). Therefore, we did not consider our endophytic strain from banana as a biocontrol candidate for agricultural applications.

*Pseudomonas aeruginosa* has been frequently isolated as endophytes from different crop plants and most of such strains have been reported to show inhibitory activity against various phyto-pathogens. These include antagonistic activity against *Phytophthora capsici* in black pepper (Aravind et al., 2009), *Sclerotium rolfsii* in cucumber (Pandey et al., 2012), *Colletotrichum gloeosporioides* in chili (Allu et al., 2014), *Pythium myriotylum* in ginger (Jasim et al., 2014), *Fusarium oxysporum* in cotton (Yasmin et al., 2014) and wheat (Gupta G. et al., 2015), *Ralstonia solanacearum* in tomato (Maji and Chakrabartty, 2014) and *Xanthomonas* sp. infecting different crops (Spago et al., 2014). Endophytic *P. aeruginosa* is also known to be an effective biocontrol agent against nematodes (Ali et al., 2002; Kumar et al., 2013). Further, some strains of *P. aeruginosa* have been reported as plant growth promoters (Pandey et al., 2012; Gupta et al., 2013; Gupta G. et al., 2015) or useful in weed management (Lakshmi et al., 2015). Thus the endophytic strain of *P. aeruginosa* from banana (Sekhar and Thomas, 2015) appeared to form a good model system for studying the twin aspects of (i), the ability of an endophytic strain to survive in soil and (ii), effects due to the introduced microorganism with pathogen-antagonistic potential on native soil microbial community. Further, *Pseudomonas* sp. represents one of the most abundant genera of the root microbiome (Zamioudis et al., 2013). The present investigations were undertaken to assess the survivability of the endophytic *P. aeruginosa* strain from banana under non-axenic conditions in agricultural soil and rhizosphere and to gauge the effects due to the introduced organism on native microbiome by monitoring the gross cultivable bacterial community as the representative indicator of soil microbiota.

## Materials and Methods

### Endophytic Bacterial Strain

Endophytic *P. aeruginosa* strain ‘GNS13.2a’ (NCBI 16S rRNA gene accession number KP798813) isolated from the deep-seated shoot-tip tissue of banana sucker cv. Grand Naine (*Musa* sp.; AAA genome) in a previous study (Sekhar and Thomas, 2015), referred to as *Pau* hereafter, was employed as the test organism in this study. The isolate was maintained as glycerol (30%) stock at -80°C and revived on nutrient agar (NA) followed by single colony perpetuation at each culturing. This strain was not specifically tested for human or animal pathogenicity. The experiments were conducted under containment followed by destruction of all biosamples through autoclaving or formaldehyde drenching. A Class-II vertical airflow cabinet with ULPA filter (Esco Biotech, Pvt. Ltd., Singapore) was employed during axenic works.

### Identification of Bacteriological Media for *Pau* Monitoring

To select the appropriate media for capturing the culturable bacterial community and for monitoring *Pau* specifically, NA was tested compared with two known *Pau* selective media. These included (i), NA containing 60 μg ml^-1^ kanamycin and 50 μg ml^-1^ 2,3,5-triphenyl tetrazolium chloride (Kan+TTC:NA) as used by Kumar et al. (2013), and (ii), cetrimide agar base added with 15 μg ml^-1^ nalidixic acid (Goto and Enomoto, 1970) as employed by Deredjian et al. (2014). The above media were tested using pure culture of *Pau*. For this, six decimal dilutions (10^1^ to 10^6^) in sterile distilled water (SDW) considering 0.1 OD_600__nm_ overnight NA colony-derived culture as 10^0^ ‘anchored stock’ (Thomas et al., 2015) were applied through SATS approach (Thomas et al., 2012). The plates were observed at 30°C for 1–4 days for *Pau* cfu. The dilution level that yielded cfu in the 30–300 range was selected based on which cfu ml^-1^ of 0.1 OD stock in different media was worked out (Thomas et al., 2015).

Further, pure culture of *Pau*, irrigation grade water and rhizospheric field soil were tested on the above three media to ascertain the suitability for harnessing *Pau* cfu specifically. Here, the six serial dilutions from *Pau* anchored stock as above were tested by spotting 20 μl aliquots side by side in six sectors in a 9-cm Petri dish, a method designated as single plate-serial dilution spotting (SP-SDS) (Thomas et al., 2015). The anchored stocks (10^0^) constituted 0.1 OD suspension for pure bacterial cultures, original sample for irrigation water and 1 g per 10 ml SDW for soil samples. Irrigation water was tested directly and after mixing with *Pau* stock. Soil sample suspensions were tested similarly after mixing with *Pau* culture employing six serial dilutions. The plates after surface drying were observed for bacterial cfu and specificity for *Pau* detection over 7 days.

Based on the results, NA was selected as the standard medium for growing pure cultures of *Pau* and for total culturable bacterial monitoring. Cetrimide-nalidixic acid-agar (CNA) was identified as the selective medium for the specific monitoring of *Pau* with obvious colony development within 24 h similar to NA. The GNS13.2a isolate had kanamycin and rifampicin resistance as genetic markers besides nalidixic acid. Modification of CNA with 60 μg ml^-1^ kanamycin was tried which delayed the colony growth and enumeration by 1 day with no extra benefit on cfu or selection specificity. Cetrimide agar with 50 μg ml^-1^ rifampcin (Matos et al., 2005) also did not offer any advantage over CNA while testing field soil or irrigation water for *Pau*. Further, 1–2 days-old NA plate cultures of *Pau* were tested directly and after refrigeration for cfu ml^-1^ on NA and CNA (six replications) to ensure that the inocula employed had good viability. All media formulations and supplements were sourced from M/s Hi Media Biosciences, Mumbai, India.

### Pre-monitoring of Irrigating Water and Soil-Mix for *Pau*

As a prerequisite to ascertain whether the water used for irrigation, or the rhizospheric soil-mix employed in pot-culture trials harbored any *Pau*, both were checked 4 days prior to the start of the soil-monitoring experiment. Piped irrigation-grade water collected and stored in an open plastic tank in the glasshouse 1 day before was generally used for watering the soil. Aliquots of 100 μl were applied directly on CNA plates (100 nos) which altogether offered a gross detection sensitivity of 0.1 cfu ml^-1^. The soil stock was comprised of a 1:1:1 blend of rhizospheric soil where banana was being gown, river sand and well-decayed farm yard manure (pH 7.35 ± 0.44) without any chemical or other sterilization treatments. The soil mix (50 g in 500 ml autoclaved water; 10^0^ stock) was shake incubated for 1 h at 200 rpm and plated at 10^1^ dilution on CNA plates (100 nos) offering a detection sensitivity of 1 cfu g^-1^ soil. The plates were monitored for microbial cfu and distinct colony types for 4 days in comparison with pure culture of *Pau*. Two colony morphotypes that emerged on CNA from water (2–4 × 10^1^ cfu ml^-1^) and soil (1–4 × 10^2^ cfu g^-1^) and distinct from *Pau* reference colonies in appearance were taken for identification through 16S rRNA gene sequence analysis as described elsewhere (Sekhar and Thomas, 2015). Mix culture of these organisms was further tested on NA and CNA along with pure *Pau* culture through SATS to ensure their distinction from *Pau* colonies.

### Monitoring of *Pau* in Inoculated versus Control Soils Relative to Native Cultivable Bacterial Biome

To monitor the survival of *Pau* in inoculated versus control soils, the rhizospheric soil mix from the same lot as above in pots was employed as it was not possible to get a clear estimate under field conditions. Plastic pots (7′′ height and 6.5′′ diameter) were provided with 2.5 kg of dry soil-mix per pot and watered to field capacity. After 24 h under glasshouse conditions, eight replicate pots were drenched with 250 ml of 0.1 OD *Pau* suspension (about 10^8^ cfu ml^-1^) from day-2 NA plate colonies (*Pau^+^* soil; approximately *Pau* cfu of about 10^7^ g^-1^) and the control pots (*Pau*^-^ soil) were applied with equal volume of SDW. First soil sampling was done on the same day (day-0) within 30–60 min. This involved inserting a 15 ml sterile Falcon tube to the soil to its full length (to a depth of approximately 10 cm) during which approximately 2 cm of compacted soil (approximately 2–2.5 g) was collected inside the tube. The sample from eight such pots was pooled and mixed thoroughly after removing the stony particles and breaking the lumps. 10 g soil was dispersed in 100 ml of SDW (10^0^ stock) in a sterilized container and *Pau* and non-*Pau* cfu were assessed through SATS employing four replications per dilution. The *Pau*^-^ soil samples were also processed similarly and monitored on NA and CNA. *Pau* colonies were located on NA based on the bluish green tinge while on CNA they appeared as shining/fluorescent cream-yellow colonies. The cumulative cfu on day-4 was used for estimating the *Pau* versus non-*Pau* cfu which was expressed as cfu g^-1^ soil. A simple assessment of the extent of bacterial variability was made on day-4 by counting the different colony types formed on NA at dilutions (10^3^ or 10^4^) that yielded well-delineated colonies.

The pots were left open under glasshouse conditions (day temperature range of 25–30°C; irradiance of 500–600 μE m^-1^ s^-1^ for 8–10 h) using 250 ml of irrigation-grade piped water per pot daily in the afternoon. Sampling of *Pau^+^* and *Pau*^-^ soils was repeated on days-4, 7, 14, 21, and 28 with sample collection during the 30–60 min time span after watering. The irrigation water sample was routinely monitored for bacterial cfu by plating on NA and CNA. The pots thereafter were left without watering allowing the soil-mix to dry completely. The survival of *Pau* under dry soil conditions was monitored as above after another 4 weeks preceded by watering 4 h before sampling. This soil was monitored for *Pau* again after 48 h.

To assess the effect due to the moistening of dry soil in altering the total culturable bacterial cfu and the contribution of irrigating water to it, a further trial was set up. The dry soil-mix (2.5 kg in plastic pots) was applied with 500 ml of (i) sterile water or (ii) irrigation-grade water (3.2 × 10^5^ cfu ml^-1^). The initial soil bacterial cfu was monitored within 30–60 min on NA. Thereafter, the pots were kept covered with polythene sheet to avoid the lateral introduction of organisms, and were monitored for bacterial cfu again after 24 h. A third treatment involved rhizospheric soil from pots planted with tomato (6 weeks post-planting). These pots were maintained under glasshouse conditions, watered daily with irrigation-grade water (250 ml) and cfu estimations were undertaken as above.

### Validation Trials Employing Rhizospheric Soil-Mix and Banana Rhizospheric Soil in Pots

A validation trial was undertaken employing a new batch of rhizospheric soil-mix watered to field capacity one day prior to *Pau* inoculation as discussed above (*Pau*^+^ and *Pau*^-^ soils) employing eight replicate pots. Another experiment was set up employing pots which were planted with tissue-cultured banana ‘Grand Naine’ saplings for the preceding 2 months (pH 7.13 ± 0.37) with daily inundation using irrigation-grade water. The *Pau*^+^ and *Pau*^-^ soils were monitored for *Pau* and gross cultivable bacterial flora on days-0, 4, and 7 employing CNA and NA after clearing the root tissues. Irrigation was practiced daily as above with periodic monitoring of water employing NA and CNA.

### Monitoring the Survival of *Pau* in Axenic Soil Culture

For this, the soil-mix from the same lot described above was employed. Dry rhizospheric soil-mix (200 g) was wetted to field capacity employing 50 ml distilled water in 300 ml volume glass bottles with wide mouth (50 mm diameter). The screw capped bottles were subjected to autoclaving for 20 min at 121°C (1.1 kg cm^-2^) on three consecutive days. On the third day, the bottles were kept open in the vertical airflow cabinet for 1 h followed by the addition of 10 ml of 1.0 OD *Pau* culture (about 10^9^ cfu ml^-1^) per bottle employing eight replications. The control bottles were provided with SDW. The soil was monitored on the same day (day-0) and thereafter on days 1, 4, 7, 14, 21, and 28 on CNA for the extent of *Pau* cfu and on NA to check for any lateral introduction of non-*Pau* cfu during samplings. The soil from eight replicate *Pau*^+^ and *Pau*^-^ bottles was collected separately in 50 ml tubes, weighed aseptically and dispersed in sterile water (1 g per 10 ml) followed by SATS of decimal dilutions on NA and CNA.

### Monitoring of *Pau* in Established Axenic Soil Culture Following Lateral Introduction of Soil Microbiome

With a view to assess the response of established *Pau* flora in axenic soil to the exposure to native soil microbiota, *Pau* population was initiated in 3× autoclaved soil-mix in glass bottles as above followed by the introduction of soil microbiome. For this, the baseline *Pau* cfu was assessed by pooling the soil from two sets of *Pau* inoculated bottles 2 weeks post-inoculation (*Pau^+^* axenic soil sets-I and II) employing four replications. The set-I was applied with 5 ml of SDW while the set-II was added with 5 ml of the supernatant from the soil-mix suspended in SDW (1 g per 10 ml). *Pau* and non-*Pau* cfu were assessed on NA after 48 h. Thereafter, the set-I was applied with 5 ml SDW while the set-II was applied with 5 ml of 1.0 OD suspension of pooled bacterial inoculum prepared from NA colonies of soil bacteria derived from the plating of soil-mix 2 days before. The bottles were kept open in the vertical airflow cabinet for 4 h to evaporate away the excess moisture, and the *Pau* versus non-*Pau* cfu was assessed on NA and CNA soon after.

### Testing the Interactive Effects of *Pau* with Soil Microbiota in Suspension

This trial was undertaken to ascertain whether the low cfu of non-*Pau* isolates on NA during the day-0 monitoring of *Pau^+^* soil arose from the antagonistic effects by *Pau* on the agar plate or due to the interactive effects in the soil itself. The soil suspensions of *Pau*^+^ soil samples from glasshouse pots prepared on days-0, 4, 7, or 14 in SDW and left under sealed conditions in 50 ml falcon tubes (to avoid the lateral introduction of microorganisms) were monitored on CNA and NA after 7 days of stationary incubation. The cfu on the date of original sampling (days-0, 4, 7, or 14) was adopted as the base reference point. As a control to test the ability of the organism to survive under static non-aerated conditions, the axenic culture of *Pau* in SDW under identical conditions was employed.

### Testing the Interactive Effects of *Pau* with Cultivable Soil Bacterial Isolates in Agar Plates

With a view to assess that the cfu reduction of *Pau* was arising from the interactive effects between *Pau* and soil bacteria, 10 random representative colony types that developed on NA from *Pau*^+^ soil during the day-0 sampling of soil in the first trial were selected. A bacterial lawn of individual soil isolates was prepared on NA by applying 100 μl of 0.1 OD inoculum in peptone-salt (Thomas et al., 2012) in 9-cm diameter plates. After allowing 1 h for the organism to establish, 25 μl of 0.1 OD *Pau* inoculum prepared in SDW was applied at the center of the agar plate (6–7 mm diameter well) followed by air-drying in the vertical airflow cabinet for 25–30 min. The reaction of *Pau* to the test isolate and *vice versa* was assessed based on (i), the diameter of spreading *Pau* colony growth from the center of the well, (ii), the extent of clear zone, if any, between *Pau* and the isolate in the lawn, and (iii), the extent to which the lawn of the test isolate was pushed to the outer edge of NA plate by *Pau*. The test plates were scored on a – to ++++ scale for the above characteristics representing none, low, medium, high or significant. Based on the pooled information, four categories of interactive effects were documented: (i), no mutual antagonism (with or without dominance by *Pau*), (ii), aggressive antagonistic effect by *Pau* on the soil isolate, (iii), significant anti-*Pau* effect by the soil isolate, and (iv), mutual antagonism. The experiment was repeated with additional 20 distinct colony types selected from NA plates that were employed for the monitoring of *Pau^+^* pot-soil on day-7 (4th day after SATS) and 20 colony types from the control soil.

A direct confrontation assay was set up where the lawn of *Pau* on NA prepared using 100 μl of 0.1 OD culture 4 h after plating was applied with 5 μl of 0.1 OD inoculums of the test organisms. Based on the outcome, preparation of *Pau* lawn on NA using 0.001 OD inoculum (based on pre-trials employing 100 μl of 0.01, 0.001, or 0.0001 OD inoculum) followed by spotting with the challenge isolate as above (5 μl of 0.1 or 1.0 OD inocula) was tried. The ability of different isolates to grow or establish on *Pau* lawn at different strengths or any clear-zone development between *Pau* and the test isolate was recorded 1–4 days from the start of the experiment.

### Observations and Statistical Analysis

The ability of *Pau* strain to survive in soil *vis-à-vis* the interactive effects on soil bacterial community were assessed primarily based on the cumulative cfu data recorded as on day-4 from the date of plating on CNA and NA. The survival of *Pau* was assessed based on the percent difference in cfu keeping the day-0 cfu in soil as the reference point. The extent of non-*Pau* cfu in *Pau^+^* soils was assessed with reference to the native bacterial cfu in *Pau*^-^ control soil samples on NA. During soil samplings, eight replications were employed for *Pau*^+^ and *Pau*^-^ treatments. For all cfu comparisons, unless mentioned differently, two independent serial dilutions were prepared from pooled soil/water samples and applied as per SATS on two plates per dilution, thus constituting four replications. For statistical analysis, the cfu data were subjected to logarithmic transformation and Single Factor ANOVA or *t*-test (assuming equal variance) using the Data Analysis package of Microsoft Excel 2010. The mean (logarithmic scale) ± standard deviation values are presented.

## Results

### Validation of Selective Medium for *Pau* Monitoring

**FIGURE 1:**
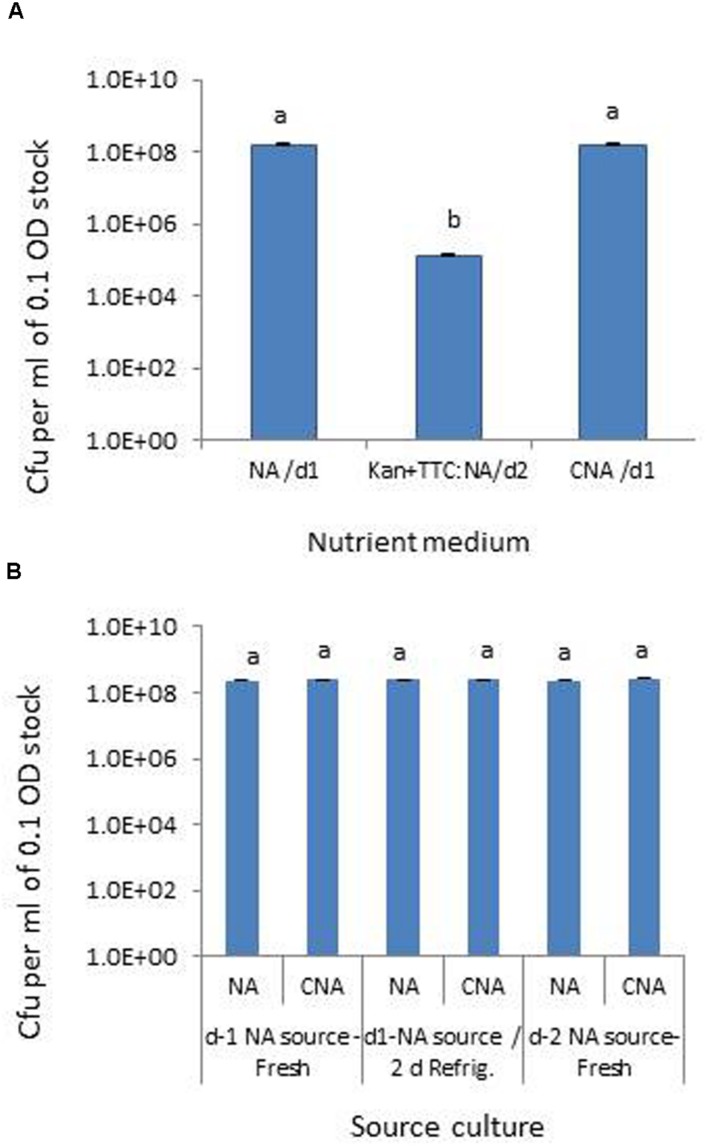
**Assessing different media for the selective monitoring of *Pseudomonas aeruginosa* (*Pau*) based on cfu ml^-1^ of 0.1 OD anchored stock **(A)** and testing of source *Pau* cultures of different ages for cell viability based on cfu **(B)**.** NA/d1 and CNA/d1 in **(A)** indicate cfu recorded on respective media on day-1 and Kan+TTC:NA/d2 cfu observed on day-2. Vertical bars indicate standard deviation. Bars with same letters are not significantly different at *P* = 0.05.

Testing the *Pau* serial dilutions on three media, NA and CNA showed colonies in the 30–300 range at 10^5^ dilution of the original 0.1 OD stock within 18–24 h while it took an extra day for colony emergence on Kan+TTC:NA with obvious cfu at 10^1^ to 10^3^ dilutions of the stock culture only. CNA gave similar cfu estimates as for NA within 24 h while Kan+TTC:NA showed significantly low estimate (*P* < 0.0001; **Figure 1A**). *Pau* colonies on NA showed a blue-green tinge and they tended to fade away after 4–5 days whereas on CNA they remained delineated with a fluorescing yellow shade for several days. A comparison of *Pau* day-1, day-2 NA source cultures versus day-1 NA plate culture refrigerated for two nights showed similar cfu estimates for different age groups on both NA and CAN (*P* = 0.104; **Figure 1B**). This endorsed the usage of day-1 or day-2 NA cultures directly or after 1–2 days of refrigeration as start culture without loss of viability.

Now, testing the different dilutions of *Pau* pure culture, irrigation-grade water and soil samples (with or without *Pau* addition) in SP-SDS on the above mentioned three media (**Supplementary Figures S1A–E**) indicated (i), similar cfu at 10^5^ dilution in NA and CNA on day-1 itself, (ii), Kan+TTC:NA required an extra day for colony growth displaying countable cfu at 10^2^ dilution, (iii), Kan+TTC:NA supported the growth of several non-*Pau* organisms from irrigation water and soil, (iv), NA displayed considerable bacterial variability considering the diverse colony types, (v), CNA did not support any water or soil isolates, (vi), *Pau*-mixed irrigation water and soil showed characteristic *Pau* type colonies on NA distinguishable from the native bacterial flora based on colony features, and (vii), CNA showed pure *Pau* colonies with cfu on par with *Pau* colony counts on NA. It was quite striking that Kan+TTC:NA supported the growth of diverse microorganisms including fungi from soil while NA normally did not support fungal growth. This was validated in a repeat trial where the irrigating water and soil suspensions were mixed with 10^2^ serial dilution of *Pau* 0.1 OD stock (data not shown). *Pau* colonies from *Pau*-added soil and water were clearly distinguishable on NA when they were not entirely masked by the native bacterial colonies at higher cfu.

### Pre-monitoring of Irrigating Water and Rhizospheric Soil-Mix for *Pau*

**FIGURE 2:**
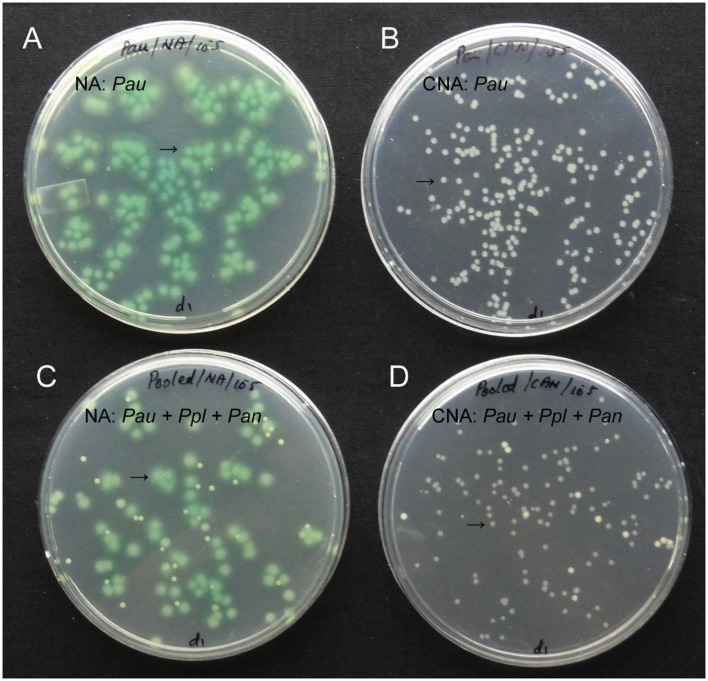
**Pure culture of *P. aeruginosa* (*Pau*) showing cfu at 10^5^ of the 0.1 OD anchored stock as bluish-green tinged larger colonies on nutrient agar (NA, **A**) or as white distinct colonies on CNA **(B)**, or a mix culture with *P. plecoglossicida* (*Ppl*) and *Pantoea ananatis* (*Pan*) showing clearly identifiable *Pau* colonies on NA **(C)** and CNA (**D**; *Pau* indicated by arrow)**.

Testing up to 10 ml water sample directly for the presence of *Pau* (100 μl in 100 CNA plates) indicated some *Pau*-unlike cfu in the range of 1–8 per 100 μl (1.0–8.0 × 10^1^ cfu ml^-1^) which was too low considering the cfu ml^-1^ of about 10^8^ for 0.1 OD *Pau* stock. The soil sample used at 10^1^ dilution showed a meager 1–4 cfu per plate (1.0–4.0 × 10^2^ cfu g^-1^ soil) which did not include any *Pau* as per the colony characteristics. There were two colony types other than *Pau* that were supported on CNA. These were identified as *Pseudomonas plecoglossicida* (predominant) and *Pantoea ananatis* (occasional). Testing the mix culture of *Pau* with the above two organisms indicated that *Pau* colonies were clearly distinguishable on NA and CNA based on the bluish green/yellow tinge and colony size by day-2 (**Figure 2**).

### Monitoring of *Pau* Inoculated versus Control Soils with Reference to Soil Bacterial-Biome

**FIGURE 3:**
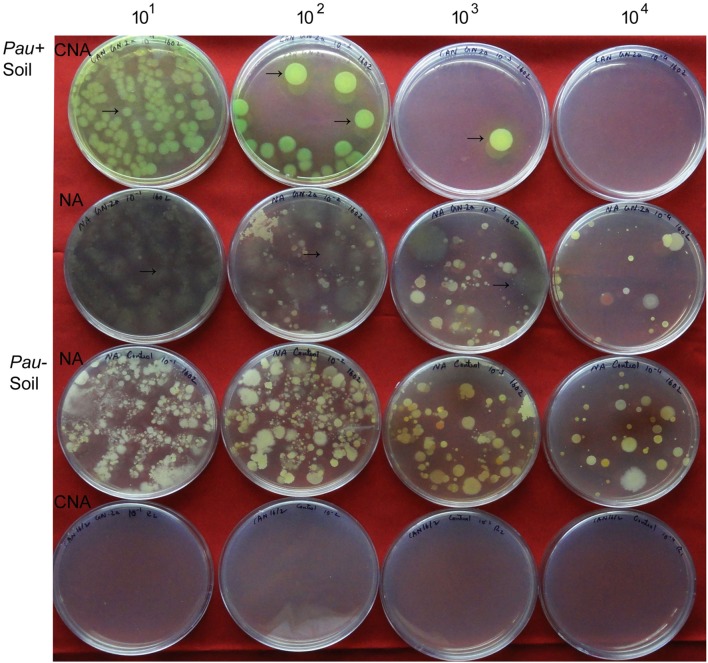
**Plating of serial dilutions (10^1^ to 10^4^) of *Pau^+^* and *Pau*^-^ rhizospheric soil mix on CNA and nutrient agar (NA) at the start of the experiment (day-0) and documentation of *Pau* (→) and non-*Pau* cfu as on day-4 after plating (row 1: *Pau* colonies from *Pau*^+^ soil on CNA; row 2: *Pau* cfu on NA appearing brownish and vanishing gradually with evident non-*Pau* bacterial cfu; row 3: *Pau*- soil showing non-*Pau* colonies on NA; row 4: no colony growth on CNA from *Pau*- soil; Colony counts were taken on days 1, 2, and 4 with the marking of initially formed ones on the reverse of the plate)**.

**FIGURE 4:**
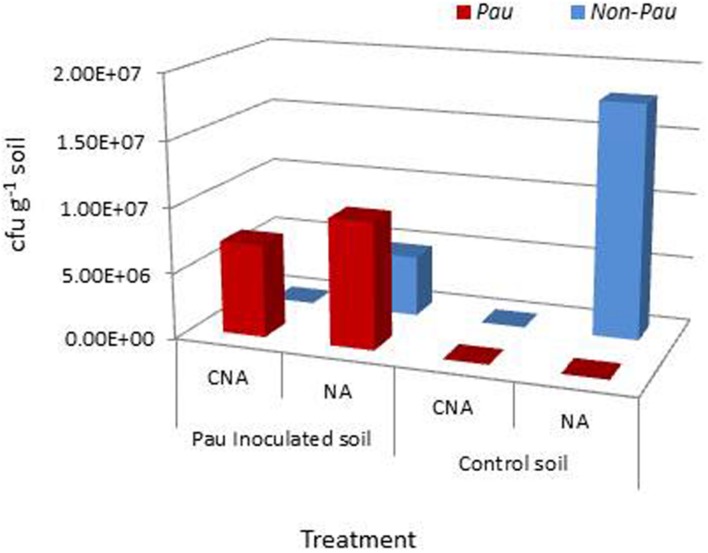
**Extent of *P. aeruginosa* (*Pau*) and non-*Pau* bacterial cfu in potted rhizospheric soil mix within 30–60 min of *Pau* application as per the cfu estimations on the selective CNA medium and NA; (cumulative of cfu recorded on day-1 to day-4)**.

The salient observations from the monitoring of *Pau* in potted *Pau^+^* soils on the date of inoculum application (**Figures 3** and **4**) included: (i) the *Pau^+^* soil yielded only *Pau* colonies on CNA to the tune of 7.1 × 10^6^ g^-1^, (ii), both *Pau* and non-*Pau* colonies were easily identified on NA registering a slightly higher *Pau* cfu (9.7 × 10^6^ g^-1^) than on CNA, (iii), no fresh *Pau* colonies emerged after day-1 on NA from *Pau^+^* soil while non-*Pau* colonies continued to emerge for 2–4 days, and (iv), the non-*Pau* cfu in *Pau^+^* soil was significantly lower (4.6 × 10^6^ g^-1^) than the corresponding value for un-inoculated control (1.8 × 10^7^ g^-1^; *P* = 0.0071). *Pau*^-^ soil did not exhibit any colony growth on CNA nor yielded any *Pau*-like colonies on NA (**Figures 3** and **4**). Beyond 4–5 days, *Pau* colonies tended to fade away or vanish from NA plates, also causing the waning of a major share of non-*Pau* colonies. Thus, the gross cfu estimate on day-4 with colony enumerations on days-1, 2, and 4 was adopted for monitoring *Pau* versus non-*Pau* cfu on NA. Marking the initially formed colonies on the reverse of the plate facilitated clearer cfu enumeration.

**FIGURE 5:**
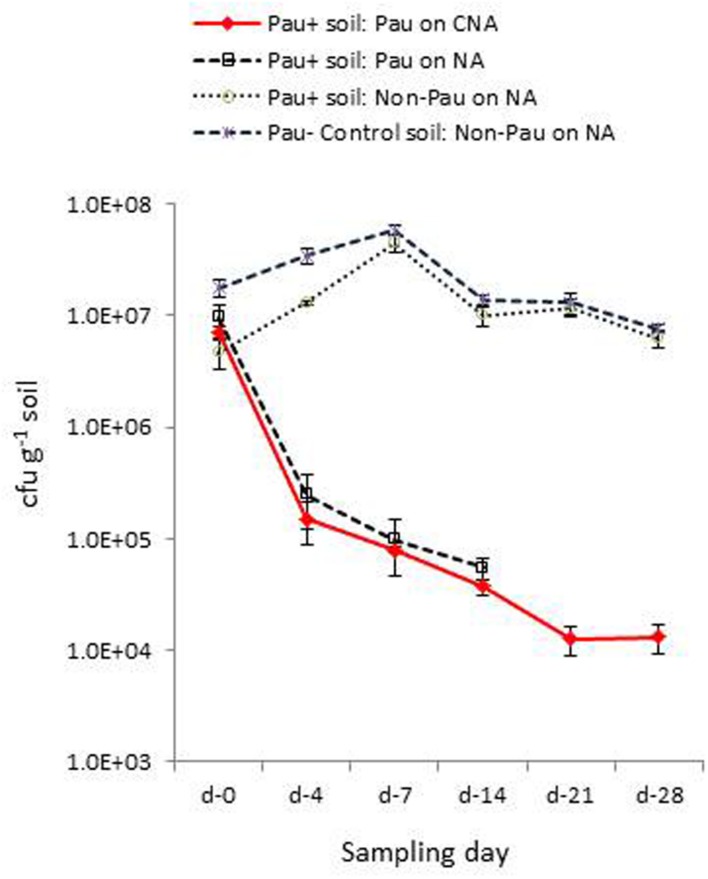
**Extent of *P. aeruginosa* (*Pau*) and non-*Pau* bacterial cfu in potted *Pau*-inoculated (*Pau^+^*) and control (*Pau^-^*) rhizospheric soil mix on different sampling dates commencing from the date of *Pau* application (day-0) as per the cfu estimations on selective CNA and NA (cumulative of cfu recorded on day-1 to day-4; vertical bars indicate standard deviation)**.

Monitoring the *Pau*^+^ and *Pau*^-^ soils in the pots kept under glasshouse conditions on day-4 indicated a significant reduction in *Pau* cfu in *Pau*^+^ soil compared with the day-0 cfu registering 2.2 × 10^5^ (-97%; *P* < 0.0001) and 2.5 × 10^5^ cfu g^-1^ (-97.4%; *P* < 0.0001) on CNA and NA, respectively (**Figure 5**). On the other hand, the non-*Pau* cfu in *Pau*^+^ soil showed a rise to 1.3 × 10^7^ g^-1^ (from day-0 cfu of 4.6 × 10^6^ g^-1^; 182%; *P* < 0.0001). This indicated a gradual build-up of native bacterial community following the initial counter effect by *Pau*. The cfu of non-*Pau* organisms on NA from control soil (3.4 × 10^7^ g^-1^) also showed a significant increase from day-0 (1.8 × 10^7^ g^-1^; 93.3%; *P* = 0.0016) to day-4 indicating a surge in native bacterial community following the watering of dry soil which was commenced 1 day prior to the start of the trial.

The day-7 sampling indicated a further reduction in *Pau* cfu from day-4 cfu in *Pau^+^* soil (7.9 × 10^4^; *P* < 0.001, and 9.8 × 10^4^ g^-1^; *P* = 0.002, respectively, on CNA and NA). The non-*Pau* cfu here showed a significant increase (4.5 × 10^7^ g^-1^) over the day-0 (4.7 × 10^6^ g^-1^; *P* < 0.001) and day-4 cfu (1.3 × 10^7^ g^-1^; *P* < 0.001) indicating native bacterial community build up after the initial attack by *Pau*. The level attained here by day-7 (4.5 × 10^7^ g^-1^) reached nearly close to that recorded for the un-inoculated soil (5.8 × 10^7^ g^-1^; *P* = 0.05). The sampling done on day-14 indicated a further drop in *Pau* population which continued through day-21 and almost stabilized by day-28 (**Figure 5**). It was not feasible to document *Pau* cfu of *Pau*^+^ soil on NA at this stage due to the too few colonies which were masked by high non-*Pau* cfu. During this phase both the *Pau^+^* and *Pau*^-^ sets also displayed a gradual reduction in non-*Pau* cfu indicating stabilization of native bacterial community to similar cfu levels in both cases.

Monitoring the *Pau*^+^ soil after another 4 weeks with an intervening 4-weeks dry spell showed barely any *Pau* (1.0 × 10^2^ to 2.0 × 10^2^ cfu g^-1^) indicating poor survival of *Pau*. The native non-*Pau* population in both *Pau*^+^ and *Pau*^-^ soils appeared comparable which was similar to the levels documented before the onset of 1 month dry spell (7.4 × 10^6^ and 7.9 × 10^6^, respectively; *P* = 0.66). The sampling 48 h after the rehydration of above soils showed a slightly more but no significant increase in *Pau* colonies in *Pau*^+^ soil (1.4 × 10^3^ cfu g^-1^). It was striking to note that the cfu of non-*Pau* organisms capable of growing on CNA showed an increase in *Pau*^+^ soil (4.0 × 10^3^ cfu g^-1^) while the *Pau*^-^ soil did not show such colonies. In other words, the non-*Pau* organisms supported on CNA appeared more rampant in *Pau*^+^ soil than in un-inoculated soil.

The irrigating water did not show any *Pau* colonies on CNA during the periodic monitoring. The control soil sample also did not yield any *Pau*-like colonies on CNA but the *Pau*^+^ soil showed a few *Pau*-unlike colonies capable of growing on CNA during the 8 weeks monitoring. Identification of these distinct colony morphotypes confirmed them to be not *Pau*; these included *P. plecoglossicida*, *P. monteilii, P. taiwanensis*, and *Achromobacter* sp. detected in very few counts on CNA (0–4 cfu) applied with the 10^1^ dilution of soil suspension. These were distinguishable from *Pau* colonies when grown together based on color and size on CNA and NA. Randomly selected supposedly *Pau*-colonies were confirmed to be so with 16S rRNA typing.

**FIGURE 6:**
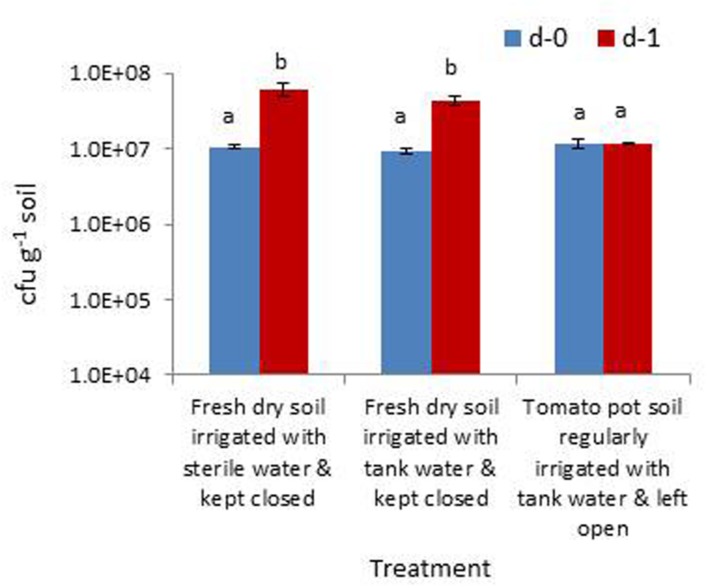
**Assessing the extent of increase in gross bacterial cfu due to the moistening of dry rhizospheric soil mix with sterile water, the contribution from irrigating water and the monitoring of rhizospheric soil of tomato (Bars with same letters are not significantly different at *P* = 0.05)**.

The trial assessing the contribution of soil wetting to the build-up of native bacterial cfu in dry soil and the relative contribution from the irrigation water indicated that the cfu hike in dry soil 24 h after the application of sterile water was mainly contributed by the activation of bacterial cells to cultivation and/or their multiplication than the direct contribution from irrigating water. This was evident from the cfu levels in the treatment inundated with irrigation-grade water (3 × 10^5^ cfu ml^-1^) which showed a relatively lower amount of cfu increase (**Figure 6**). The rhizospheric soil of tomato showed identical cfu during day-0 and day-1 samplings indicating a microbially buffered condition therein.

### Validation Trials Employing Rhizospheric Soil-Mix and Pots with Banana

**FIGURE 7:**
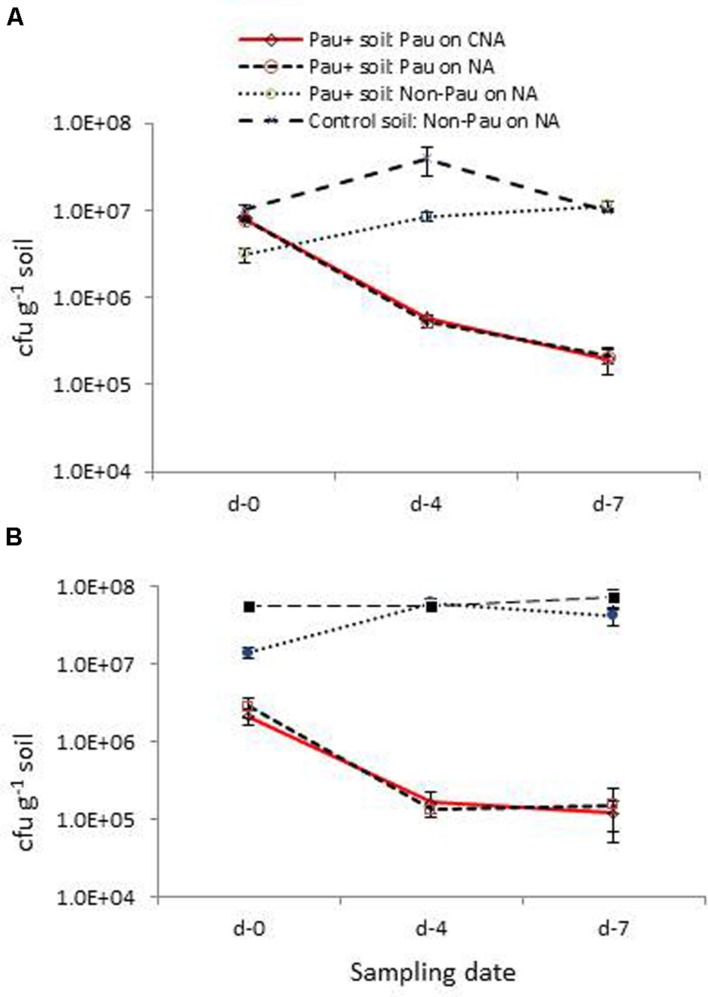
**Validation trials employing fresh field soil **(A)** and micro-flora stabilized banana rhizospheric soil **(B)**: extent of *P. aeruginosa* (*Pau*) and non-*Pau* organisms in potted *Pau*-inoculated (*Pau^+^*) and control (*Pau^-^*) field soils on different sampling dates commencing from the date of *Pau* application (day-0) as per the cfu estimations on CNA and NA (cumulative of cfu recorded on day-1 to day-4; vertical bars indicate standard deviation)**.

The low survival of *Pau* in field soil and the adverse effect on soil bacterial community were verified in this repeat trial where the dry soil was watered to field capacity 1 day prior and applied with the *Pau* inoculum/sterile water as discussed above (**Figure 7A**). Monitoring of soil on day-0, day-4, and day-7 for *Pau* and non-*Pau* bacterial cfu on CNA and NA endorsed that (i), *Pau* was less fit to survive in soil, (ii), *Pau* application disturbed the native bacterial community instantly and (iii), the soil bacterial community showed a revival following the reduction in *Pau* population.

The parallel trial employing pots which were planted with banana ‘Grand Naine’ plants showed a similar pattern of significant reduction in *Pau* cfu within a weeks’ time as in the trial employing dry soils (**Figure 7B**). The native bacterial cfu in *Pau*^+^ soil also appeared significantly low compared with the respective figure for *Pau*^-^ soil initially and then showed a gradual cfu build up as observed earlier. One striking observation in this trial was a stable gross bacterial cfu for *Pau*^-^ rhizospheric soil from day-0 to day-7 in line with the observations on tomato rhizosphere soil indicating a stabilized condition when watering was practiced on a regular basis.

Monitoring of irrigation water confirmed that there was no lateral introduction of *Pau*. On the other hand, a significant share of non-*Pau* cfu and considerable diversity (25–30 distinct colony types) were observed in irrigation water. This explained the inoculum and diversity build-up in *Pau*^+^ soil after the initial adverse effect on native bacterial community by *Pau*. While the direct piped water from the irrigation source tank showed 4.3 × 10^4^ to 7.6 × 10^4^ cfu ml^-1^ with ≥15–20 diverse colony types, the stored water in the glasshouse in the open tank showed 1.3 × 10^5^ to 3.2 × 10^5^ cfu ml^-1^ with ≥25–30 colony types. This explained to some extent the variations in the non-*Pau* cfu and diversity detected in the *Pau*^+^ and *Pau*^-^ soils on some sampling dates.

### Monitoring of *Pau* under Axenic Soil Culture

**FIGURE 8:**
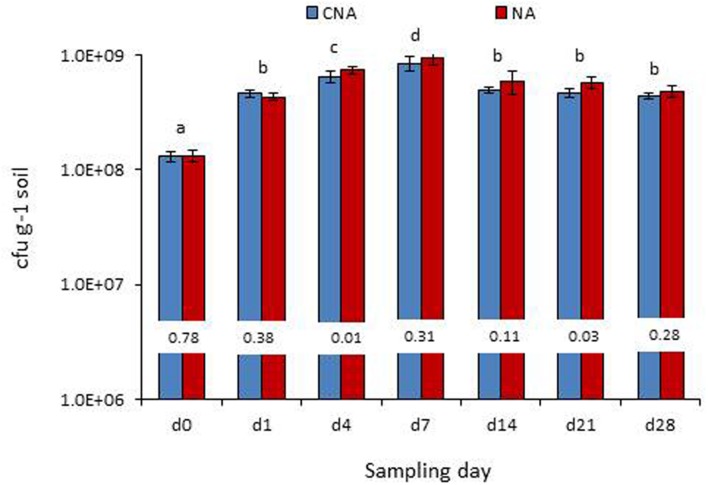
**Monitoring of the *P. aeruginosa* (*Pau*) inoculated soil under axenic conditions (bottles which were maintained under axenic conditions) employing the selective CNA and NA on different dates from the initial inoculation (Bars with same letters are not significantly different at *P* = 0.05; Values on the bar indicate *P*-value at 0.05 between the *Pau* cfu on CNA and NA)**.

On the date of inoculation of axenic soil with *Pau*, CNA and NA registered 1.30 × 10^8^ and 1.33 × 10^8^ *Pau*-cfu g^-1^, respectively, (*P* > 0.05). During the monitoring over the next 1 week, a significant increase in *Pau* cfu was observed under the axenic conditions as monitored on CNA and NA (**Figure 8**). This amounted to 2.5×, 4.0×, 5.5× increase over the day-0 base cfu as on day-1, day-4, and day-7, respectively, on CNA (*P* < 0.001 in all instances on both CNA and NA). Thereafter, *Pau* cfu showed a gradual dip but the population was still higher than that of the base level (2.8×, 2.6×, and 2.4× on day-14, day-21, and day-28, respectively). The corresponding figures while monitoring the samples on NA were 2.3×, 4.5×, 6.0×, 3.4×, 3.3×, and 2.7×, respectively, over the day-4 cfu. The un-inoculated control soil did not show any colony growth ensuring the aseptic conditions following 3× autoclaving.

### Monitoring of *Pau* in Established Axenic Soil with Lateral Introduction of Soil Microbiome

Both the sets of bottles (axenic-I and II) displayed similar *Pau* cfu at the start of the experiment (**Table 1**). The monitoring 2 days after the addition of SDW to set-1 showed an increase in *Pau* cfu by 4.6× over the cfu at the start of the experiment while set-II applied with soil suspension showed only 3.2× hike over control which was significantly low compared with the *Pau^+^* soil. The non-*Pau* cfu was not assessable as the incorporation of soil suspension added only an estimated 2 × 10^4^ cfu g^-1^ which was masked by *Pau* during the monitoring on NA plates. The monitoring of soil 4 h after the addition of NA-grown soil inoculum showed a 50% reduction in *Pau* cfu over the pre-application cfu (with the detection of a high non-*Pau* cfu). On the other hand, SDW-applied control set under axenic conditions showed a 43% increase in *Pau* cfu over the weighted average. The results overall indicated an adverse effect on established *Pau* cfu with the laterally introduced soil microbiome.

**Table 1:** Effect due to the lateral introduction of soil microbial suspension or soil derived bacterial inoculum on the population of *Pseudomonas aeruginosa* established in axenic soil for 2 weeks^a^.

Description of treatments/observations	Axenic set-I^a^	Axenic set-II^a^	Significance^b^
Base *Pau* population on NA after 2 weeks under axenic conditions (cfu g^-1^)	*Pau* cfu: 4.0 × 10^7^	*Pau* cfu: 4.5 × 10^7^	NS (*P* = 0.1619)
*First treatment imposed*	Applied with 5 ml sterile water, closed, and kept under ambient	Applied with 5 ml supernatant of soil suspension @ 1 g 10 ml^-1^ (4 × 10^6^ cfu /5 ml; final 2 × 10^4^ cfu g^-1^ bottled soil)	–
Cfu g^-1^ soil 48 h post-application of water or soil suspension	*Pau* cfu: 18.6 × 10^7^	*Pau* cfu: 14.6 × 10^7^ Non-*Pau* cfu: Too low for detection at the dilutions for *Pau* cfu enumeration	** (*P* = 0.004)
*Second treatment imposed*	Applied with 5 ml sterile water to 200 g soil	Applied with 5 ml of 1.0 OD suspension of pooled inoculum from plate grown colonies 2 days after plating the soil suspension (estimated non-*Pau* cfu: 10^9^ ml^-1^; 2.5 × 10^7^ g^-1^ soil)	
Cfu g^-1^ soil 4 h post-second treatment (bottles kept open for 4 h in the vertical air-flow cabinet before sampling)	*Pau* cfu: 28.9 × 10^7^	*Pau* cfu: 6.9 × 10^7^ Non-*Pau* cfu: 1.7 × 10^7^	** (*P* = 1.7 × 10^-6^)

*^a^Bottles with 200 g wet soil were autoclaved thrice followed by the addition of 10 ml of 1.0 OD *P. aeruginosa* suspension in SDW and incubated under ambient conditions for 2 weeks with screw cap closure. Cfu assessments were made on NA and expressed as cfu g^*-1*^ soil.*

^b^Significance with reference to difference in *Pau* cfu between axenic sets-I and II; ** significant at *P* = 0.05; NS, not significant.

### Testing the Interactive Effect with Soil Microbiota in Suspension

**FIGURE 9:**
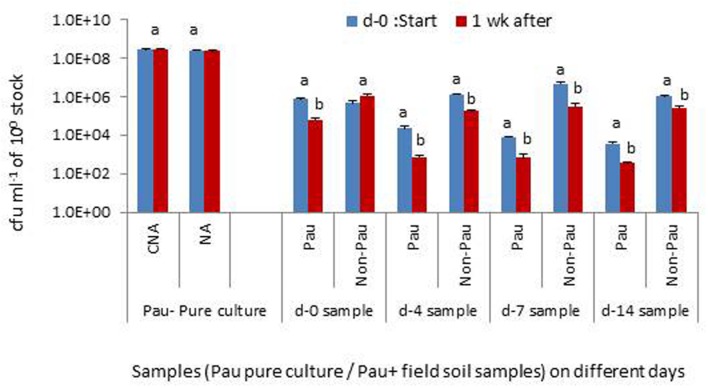
**Testing the interactive effect of *P. aeruginosa* (*Pau*) with soil microbiota in suspension employing the soil suspensions prepared on days 0, 4, 7 or 14, with static incubation for 1 week after the preparation, in comparison with the pure culture employing CNA for *Pau* and NA for non-*Pau* estimations (10^0^ stock refers to 0.1 OD anchored stock for the pure culture and 1 g per 10 ml for the soil suspension; Bars with different letters in a treatment set are significantly different at *P* = 0.05)**.

The monitoring of the soil suspensions prepared on days-0, 4, 7 or 14, a week later indicated that *Pau* was counteracted by other organisms in the suspension significantly affecting its population levels (**Figure 9**). Soil suspension also offered the entire soil microbiome including bacterial, fungal, and protozoan mircocosms in cultivable and non-cultivable forms. The monitoring of pure *Pau* suspension under identical axenic conditions indicated that the organism was capable of surviving stationary incubation with no change in cfu. The non-*Pau* community showed a revival after the initial attack by *Pau* in the sample prepared on day-0. The suspensions from days-4, 7, and 14 sampling showed a less fitness for the survival of both *Pau* and non-*Pau* sets.

### Testing the Interactive Effects of *Pau* with Soil Isolates in Agar-Plate Assay

The control NA applied with *Pau* in the agar-well at the center of the plate showed growth covering almost 30–35 mm with a plate radius of 45 mm over 3–4 days. In dual culture plates, the growth of *Pau* varied from nil or negligible around the pit to quite active growth to the extent of 15–20 mm radius as documented after 3–4 days of application. A clear-zone was evident between *Pau* and the soil isolates in most instances. The extent of lawn formed by the soil strains in dual culture plates also varied significantly depending on the isolate ranging from full growth reaching the pit domineering over *Pau* to the extent of being pushed to the outer periphery. In extreme cases, there was no obvious lawn development at all. Based on the above observations, four different categories of responses were documented: (i), null effect, (ii), severe antagonistic effect by *Pau* on the soil isolate, (iii), severe anti-*Pau* effect displayed by the soil isolate, and (iv), mutual antagonistic effects (**Supplementary Table S1**).

The spot application of soil isolates on *Pau* lawn formed on NA after plating the 0.1 OD culture and allowing 1 h for *Pau* to establish wholly inhibited the growth of test isolates. No obvious colony growth was observed from the applied spots even after 3–4 days in all the 50 test isolates. Reducing the *Pau* cell population employing 0.1, 0.01, or 0.001 OD inoculums also showed proper lawn development. Use of 0.001 OD *Pau*-inoculum for lawn plating followed by immediate spotting of soil isolate using 0.1 or 1.0 OD inoculum showed the growth of the test organism at the applied spots at 1.0 OD in a few instances but not at 0.1 OD (**Supplementary Table S1**). Thus, the presence of *Pau* even at lower cell densities before the addition of challenge isolate appeared to inhibit their establishment under the nutrient rich conditions in the plates.

## Discussion

The present investigations have brought out valuable information on the effects due to the introduction of an agriculturally significant bacterium, *P. aeruginosa*, possessing multiple pathogen-antagonism potential on the native soil microbial community and the response of the soil microbiome to the introduced microorganism. Besides, it has added the much desired information on the survivability of an endophytic strain artificially inoculated into the field soil which is generally lacking. For an organism to be effective as a biocontrol or growth promoting agent in agriculture, it should be able to sustain under field soil-conditions in sufficient populations for a minimum duration (van Veen et al., 1997). It should not disturb the native microbial community drastically which otherwise be playing diverse significant roles in the rhizosphere (Hartmann et al., 2009; Trabelsi and Mhamdi, 2013). Contrary to this, *P. aeruginosa* proved to be not a competitive inoculant for soil and its application in agricultural soil instantly affected the non-target organisms, thereby disturbing the soil microbial community dynamics. The effect, however, was transient as the non- *P. aeruginosa* cfu showed restoration on par with control soil within 1 week. This perhaps was contributed by the multiplication of survivors or the addition of new organisms through water/air, with the reduction in the population of the inoculant. The present trials were carried out in pot cultures considering the feasibility of monitoring the inoculant and the soil microbiome unlike the open field but employing the rhizospheric soil with the natural soil associates.

Studies exploring the effects due to the introduced organism on soil bacterial or microbial community depict varying conclusions depending on the organism or the soil type (Herschkovitz et al., 2005; Chowdhury et al., 2013; Kröber et al., 2014; Schreiter et al., 2014; Gupta R. et al., 2015). Most of the above studies have relied on molecular approaches/metagenomics rather than cultivation which on one hand gave a wider community impact effect but did not involve verification with direct challenge assays. Both cultivation-based and cultivation-independent approaches have inherent advantages and disadvantages. In this report, the cultivable fraction was analyzed as a whole representative of the soil microcosm and it facilitated the documentation of four categories of interaction effects varying from severe antagonistic effect by *P. aeruginosa* on the soil isolate, severe anti- *P. aeruginosa* effect displayed by the soil isolate or mutual antagonistic effects to nil effects. We did not target the identification of different isolates as the soil and irrigation water contained multitude of organisms, the cultivable fraction constitutes a minor fraction of the microbial community and that the soil microbial communities vary with location and time.

*Pseudomonas aeruginosa* was employed in this study as a model system considering the wide range of antagonistic activity reported against various pathogens including bacteria (Maji and Chakrabartty, 2014; Spago et al., 2014) and fungi (Aravind et al., 2009; Allu et al., 2014) and pests like nematodes (Ali et al., 2002; Kumar et al., 2013). The bacterium is known to produce different antimicrobial compounds which include mainly phenazines (Anjaiah et al., 1998; Mavrodi et al., 2006; Jasim et al., 2014). The putative biocontrol agent exerted significant adverse effects on non-target native microbiome at variance from the conventional wisdom of nurturing the soil microbiome. It is likely that such responses vary with the aggressiveness of the inoculants or the extent of antimicrobial activity. Considering the spectrum of antagonistic effects by *P. aeruginosa* cited above, it could be viewed as an aggressive species to which the soil community perhaps needed to respond likewise. This proposal is hypothetical at this stage but forms an aspect for future research. The observations in this study emphasize the need for pre-testing the bio-inoculant for possible adverse effects on native microbial community before recommending it as a potential biocontrol agent, particularly for organisms with broad anti-microbial activity.

*Pseudomonas aeruginosa*, in this study, proved to be a poor survivor in agricultural field soil. The sustenance and proliferation of the strain under gnotobiotic conditions but poor survival in field soil suggested that this was not a mere fitness issue but mostly due to the antagonistic effects by the native microbial community. This was endorsed by the observations from the lateral introduction of soil-microbiome in *Pau*-established axenic soil and the interactive effects of *Pau* in soil suspension both of which involved the gross microbial community including cultivable and non-cultivable microorganisms. The direct confrontation assays in agar plates also endorsed the significant role of microbe–microbe interactions though it involved a representative group of cultivable environmental bacteria. The soil microbial community includes bacteria, fungi, and viruses. The introduced organism faces challenge due to the antagonistic effects or competition from native microorganisms and is vulnerable to predation by protozoa which could affect its survivability (England et al., 1993; van Veen et al., 1997; Trabelsi and Mhamdi, 2013; Tyc et al., 2014, 2015).

Besides the above biotic factors, the environmental fitness of the introduced organisms in soil is also influenced by several abiotic factors such as soil type, pH, temperature, water content, periodicity of wetting and drying, and soil constituents including organic carbon and mineral nutrition (Acea et al., 1988; van Veen et al., 1997; Bakker et al., 2013; Trabelsi and Mhamdi, 2013; Tyc et al., 2014, 2015). The inoculant is also vulnerable to desiccation. Considering that the same soil-mix was employed for axenic studies and that *P. aeruginosa* monitoring in field soil was carried out in pot trials under glasshouse conditions where there was a better control over watering and edaphic factors, the observations suggest that the poor survival of *P. aeruginosa* under field conditions was governed by interactions with the soil microbiome rather than due to environmental factors. The situation in the field may be still harsher with alternating spells of wetting and drying or prolonged dry/wet spells compared to the controlled conditions in the glasshouse.

The low fitness of *P. aeruginosa* strain employed in this study did not appear to arise from its endophytic origin as the same has been reported with environmental strains too. Weir et al. (1996) observed a 100-fold reduction in the cfu of *P. aeruginosa* in dry soil inoculated with rifampicin-resistant environmental strain UG2Lr over 3 weeks in comparison with cells encapsulated in dry alginate beads before application. A significant interaction effect between *P. aeruginosa* and the soil community was documented in wheat rhizosphere that the invasibility was inversely related to the level of native microbial diversity (Matos et al., 2005). Adopting cultivation-based as well as molecular monitoring, *P. aeruginosa* was not detected in most native agricultural soils but sparsely observed in manure-amended soils although the bacterium showed good survival in organic-manures (Deredjian et al., 2014). Further, employing clinical and environmental strains of *P. aeruginosa*, the introduced organism showed a decline to below detectable levels after 3–5 weeks under non-sterile microcosms while the population was maintained high under sterilized microcosms (Deredjian et al., 2014). It may be argued that an endophytic strain is more suitable for plant colonization. The ‘GNS.13.2a’ strain showed colonization of banana roots. However, it is imperative that endophytic microorganisms go through a phase in the soil or environment at the end of the life of the plant or the organ. Besides, soil drenching of inoculum constitutes the best form of application toward biocontrol of soil-borne pathogens. Decline in the populations of introduced bacteria in soils have been documented for a host of organisms irrespective of the source of their original isolation (van Veen et al., 1997). The form in which endophytes survive intra-plant or in soil may be different from the nutrient rich monoculture during *in vitro* growing.

There is a general criticism that the significant pathogen and pest antagonistic effects displayed by the test organisms in the laboratory trials are not always translated to successful biocontrol strategies in the field (Acea et al., 1988; van Veen et al., 1997; Upreti and Thomas, 2015). In effect, the application of *Pau* in field soil did not serve any net beneficial effect in terms of its sustained survival. It rather caused an unwarranted transient disturbance in the soil community dynamics which otherwise was at harmony as observed with the undisturbed banana and tomato control rhizospheric soils. The population of surviving *Pau* in field soil applied with nearly 10^7^ cfu g^-1^ reached barely 0.01–0.025% of the initial population within 1 week in the instance of immediately wetted soil, and about 0.05% level for the microbially buffered banana rhizospheric soil. Such low population level in soil would perhaps be insufficient to offer a formidable and sustained protection against pathogens which survive in the field even under harsh conditions.

It is pertinent to mention that a vast majority of trials recommending the use of pest/pathogen antagonistic organisms are based on mere laboratory assays with no systematic monitoring of the survival of the organism or the effect due to the introduced organism on the native microorganisms. The observations here suggest the need for investigations on soil survival and microbe–microbe interaction effects before recommending the commercial adoption of bio-inoculants as the preservation and nurturing of native flora is also important in sustainable agriculture. The significance of microbe–microbe interactions under field conditions has not received much attention which is being gradually recognized now (Trabelsi and Mhamdi, 2013; Tyc et al., 2014, 2015). This also calls for the detailed analysis of microbe–microbe interactions while formulating microbial consortia rather than mere compatibility testing in nutrient plates and also testing the combined effect of the microbial consortium on soil microbiome.

*Pseudomonas aeruginosa* strains are known to be a human pathogen (Alonso et al., 1999; Morita et al., 2014). Therefore, it does not form a choice candidate toward biocontrol or plant growth promotion applications unless it is vividly established that the isolates infecting humans or animals and the plant/soil associated isolates are different and the possibility of horizontal gene transfers are low. The study by Kumar et al. (2013) showed the uniqueness of the endophytic isolate from pepper from other clinical isolates but the former also possessed the virulent genes as the clinical strains. A high degree of genomic conservation between *P. aeruginosa* isolates from diverse environments including clinical strains has been documented which raises concerns about the usage of this organism in agriculture (Grosso-Becerra et al., 2014). Therefore, authentic information on the relatedness between the plant isolates and human opportunistic pathogenic isolates, effect on native endophytes and the possible transmission to different plant parts all need to be considered before recommending *Pau* in agricultural applications. *P. aeruginosa* is also reported as a plant pathogen (Walker et al., 2004).

Reports highlighting the benefits of *P. aeruginosa* in terms of plant growth promotion and antagonistic effects on phytopathogens and pests and recommending the organism in agriculture are continuing to emerge as per the recent research publications cited. These studies do not mention about the monitoring of the survival and the effects on native microflora nor do they consider the potential hazards to human or animal systems. Whether the beneficial effects arise from the endophytic colonization by *P. aeruginosa* and any consequential effects on the endophytic microbiome need further investigations. The potential of transmission of the inoculant to banana fruits which is consumed in fresh form is another aspect for future research. Such investigations would be strengthened with the adoption of metagenome based analyses (Knief, 2014) facilitating interaction studies between the introduced strain and native microbiome covering cultivable and non-cultivable communities.

## Conclusion

*Pseudomonas aeruginosa* proved to be a poor survivor in agricultural soil with a quick decline in the cfu of the inoculant within a week while it showed survival and proliferation under axenic conditions. Its application in the rhizospheric soil caused an unwarranted disturbance to the native soil bacterial community which in turn fought back and showed restoration of population on par with the control soil within this time span. The observations explain the cause of poor translation of some of the laboratory results ascribable to the poor survival of the inoculant in agricultural soil and highlight the need for monitoring the sustenance and performance of the introduced organism under field soil conditions which would be different from the nutrient-rich conditions during *in vitro* assays. It also pinpoints the essentiality to assess the interactive effects of the bio-inoculant with native microbial community and the plausible adverse effects on resident soil flora before commercial recommendation.

## Author Contributions

The experiments were planned and executed together by PT and AS. PT undertook the data analysis, interpretation, and manuscript preparation. This publication bears the Institute contribution No. 38/2015.

## Conflict of Interest Statement

The authors declare that the research was conducted in the absence of any commercial or financial relationships that could be construed as a potential conflict of interest.

## Supplementary Material

FIGURE S1 **Testing of media formulations for the selective monitoring of *Pseudomonas aeruginosa* (*Pau*) by spotting six serial dilutions of the socks in a SP-SDS format employing pure culture of *Pau***(A)**, irrigation water **(B)**, field soil **(C)**, irrigation water mixed with *Pau***(D)** and field soil sample mixed with *Pau***(E)**.** NA, Kan+TTC NA and CNA represent nutrient agar, formulation as per Kumar et al. (2013) and CNA as per Goto and Enomoto (1970), respectively; f > indicates fungal colony growth.

TABLE S1 **Testing the interactive effects between banana endophytic strain of *Pseudomonas aeruginosa* with soil bacterial isolates in agar plate assays**.

## Abbreviations

CNA: Cetrimide- nalidixic acid- agar medium
Kan+TTC:NA medium: NA containing kanamycin and 2,3,5-triphenyl tetrazolium chloride
NA: nutrient agar
*Pau*: *Pseudomonas aeruginosa* banana strain ‘GNS13.2a’
SATS: spotting- and- tilt- spreading
SDW: sterile distilled water
SP-SDS: single plate – serial dilution spotting.
