# Supplementary figures and images for: Effects Due to Rhizospheric Soil Application of an Antagonistic Bacterial Endophyte on Native Bacterial Community and Its Survival in Soil: A Case Study with Pseudomonas aeruginosa from Banana
Source: Front Microbiol. 2016 Apr 26;7:493. doi: 10.3389/fmicb.2016.00493 (PMC4844927; doi:10.3389/fmicb.2016.00493)

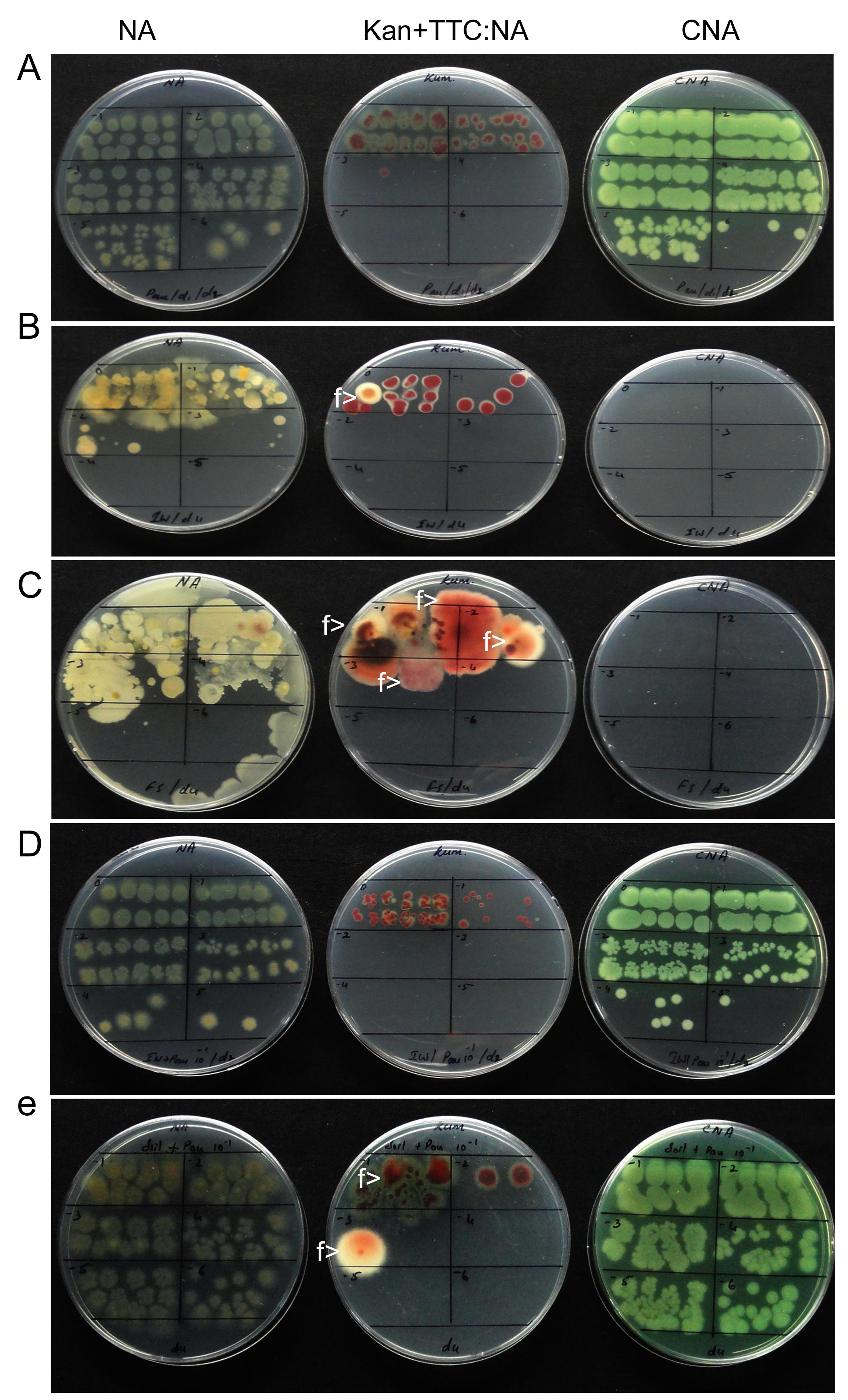

Supplement: FIGURE S1 — Testing of media formulations for the selective monitoring of Pseudomonas aeruginosa (Pau) by spotting six serial dilutions of the socks in a SP-SDS format employing pure culture of Pau(A), irrigation water (B), field soil (C), irrigation water mixed with Pau(D) and field soil sample mixed with Pau(E). NA, Kan+TTC NA and CNA represent nutrient agar, formulation as per Kumar et al. (2013) and CNA as per Goto and Enomoto (1970), respectively; f > indicates fungal colony growth. [file Image_1.JPEG]
