# Supplementary material for: Effects Due to Rhizospheric Soil Application of an Antagonistic Bacterial Endophyte on Native Bacterial Community and Its Survival in Soil: A Case Study with Pseudomonas aeruginosa from Banana
Source: Front Microbiol. 2016 Apr 26;7:493. doi: 10.3389/fmicb.2016.00493 (PMC4844927; doi:10.3389/fmicb.2016.00493)
Supplement: TABLE S1 — Testing the interactive effects between banana endophytic strain of Pseudomonas aeruginosa with soil bacterial isolates in agar plate assays. [file Table_1.DOC]

**TABLE S1.**Testing the interactive effects between banana endophytic strain of *Pseudomonas aeruginosa* with soil bacterial isolates in agar plate assays.

| Isolate ID | Gram type | Pit inoculation of *Pseudomonas aeruginosa* on the lawn of soil isolate and scoring based on growth and antagonistic effects | | | | | | | Growth of soil isolate on *Pau* lawn after spotting (5 μl of 1.0 OD inoculum) and inhibition zone development | | | | |
| --- | --- | --- | --- | --- | --- | --- | --- | --- | --- | --- | --- | --- | --- |
|  |  | Growth of *Pau^a^* | | Scoring based on clear zone*^a^* | | Depth of  lawn  growth (mm) *^a^* | | Remarks /classification based on antagonistic response | 0.1 OD lawn of *Pau* | | 0.001 OD lawn of *Pau* | | |
|  |  |  |  |  |  |  |  |  | Growth | Inhibition zone | Growth | | Inhibition zone |
| a. Day 0 isolates from *Pau*+ soil selected on day 4 from NA plates | | | | | | | | | | | | | |
| 1 | +ve | + | | - | | 33 | | Anti-*Pau* effect | - | - | | ++ | - |
| 2 | +ve | ++ | | - | | 32 | | Anti-*Pau* effect | - | - | | + | - |
| 3 | -ve | +++ | | - | | 30 | | Merging; No inhibition; *Pau* dominance | - | - | | - | - |
| 4 | -ve | ++++ | | - | | 28 | | Merging; No inhibition; *Pau* dominance | - | - | | - | - |
| 5 | +ve | +++ | | - | | 32 | | Merging; No inhibition; *Pau* dominance | - | - | | - | - |
| 6 | +ve | +++ | | + | | 24 | | Mutual antagonism | - | - | | - | - |
| 7 | +ve | - | | - | | 24 | | Strong mutual antagonism | - | - | | + | - |
| 8 | -ve | + | | - | | 40 | | Merging; Isolate domineering | - | - | | - | - |
| 9 | -ve | - | | - | | 42 | | Total suppression of *Pau* | - | - | | + | - |
| 10 | +ve | ++++ | | - | | 30 | | Merging; *Pau* dominance | - | - | | - | - |
| b. Day 7 Isolates from Pau+ soil selected on day 4 from NA plates | | | | | | | | | | | | | |
| 1 | -ve | ++++ | | - | | 20 | | Mutual antagonism | - | - | | - | - |
| 2 | +ve | +++ | | - | | 24 | | *Pau* dominance | - | - | | + | - |
| 3 | +ve | +++ | | - | | 32 | | Merging; *Pau* dominance | - | - | | + | - |
| 4 | +ve | +++ | | - | | 23 | | Strong mutual antagonism | - | - | | + | - |
| 5 | -ve | ++++ | | - | | 27 | | Strong *Pau* dominance | - | - | | + | - |
| 6 | -ve | ++++ | | - | | 12 | | Strong mutual antagonism | - | - | | + | - |
| 7 | -ve | + | | - | | 24 | | Mutual antagonism | - | - | | + | - |
| 8 | -ve | ++++ | | - | | 24 | | Mutual antagonism | - | - | | - | - |
| 9 | -ve | ++++ | | - | | 32 | | Significant antagonism on the isolate | - | - | | + | - |
| 10 | +ve | + | | - | | 30 | | Anti-*Pau* effect by the isolate | - | - | | + | - |
| 11 | -ve | ++ | | +++ | | 22 | | Strong mutual antagonism | - | - | | + | - |
| 12 | +ve | ++++ | | ++++ | | 12 | | Strong mutual antagonism | - | - | | + | - |
| 13 | -ve | ++++ | | + | | 20 | | Strong *Pau* dominance | - | - | | - | - |
| 14 | -ve | ++++ | | + | | 25 | | Strong *Pau* dominance | - | - | | - | - |
| 15 | +ve | - | | ++ | | 26 | | Strong antagonism on *Pau* | - | - | | ++ | - |
| 16 | -ve | +++ | | +++ | | 22 | | Strong mutual antagonism | - | - | |  | - |
| 17 | +ve | ++ | | ++ | | 30 | | Strong mutual antagonism | - | - | | + | - |
| 18 | +ve | +++ | | ++ | | 24 | | Strong mutual antagonism | - | - | | + | - |
| 19 | -ve | +++ | | ++ | | 24 | | Strong mutual antagonism | - | - | | - | - |
| 20 | -ve | + | | + | | 32 | | Anti-*Pau* effect | - | - | | - | - |
| c. Day 7 Isolates from control soil selected on day 4 from NA plates | | | | | | | | | | | | | |
| 1 | +ve | | + | | ++ | | 36 | Mutual antagonism | - | - | | ++ | - |
| 2 | -ve | | +++ | | ++ | | 20 | Mutual antagonism | - | - | | - | - |
| 3 | -ve | | +++ | | +++ | | 18 | Strong mutual antagonism | - | - | | - | - |
| 4 | -ve | | + | | - | | 38 | Strong anti-*Pau* | - | - | | - | - |
| 5 | -ve | | ++ | | - | | 32 | Anti-*Pau* effect | - | - | | - | - |
| 6 | -ve | | ++++ | | - | | 29 | Strong *Pau* dominance | - | - | | - | - |
| 7 | +ve | | + | | +++ | | 28 | Strong antagonism by isolate | - | - | | + | - |
| 8 | +ve | | - | | ++ | | 34 | Strong antagonism by isolate | - | - | | + | - |
| 9 | -ve | | +++ | | + | | 30 | *Pau* dominance | - | - | | - | - |
| 10 | +ve | | + | | ++ | | 30 | Anti-*Pau* effect | - | - | | + | - |
| 11 | -ve | | + | | +++ | | 28 | Strong antagonism by isolate | - | - | | - | - |
| 12 | +ve | | +++++ | | + | | 18 | Strong *Pau* dominance | - | - | | - | - |
| 13 | -ve | | +++ | | + | | 32 | Merging; *Pau* dominance | - | - | | - | - |
| 14 | -ve | | ++++ | | - | | 25 | Merging; *Pau* dominance | - | - | | - | - |
| 15 | +ve | | +++ | | ++ | | 25 | Strong mutual antagonism | - | - | | - | - |
| 16 | -ve | | ++++ | | + | | 18 | Strong *Pau* dominance | - | - | | - | - |
| 17 | -ve | | ++ | | - | | 35 | Merging; Isolate dominance | - | - | | - | - |
| 18 | +ve | | ++ | | +++ | | 24 | Strong antagonism by isolate | - | - | | - | - |
| 19 | +ve | | +++ | | ++ | | 20 | Mutual antagonism | - | - | | - | - |
| 20 | -ve | | ++++ | | + | | 18 | Strong *Pau* dominance | - | - | | - | - |

*^a^*Scoring based on Growth of *Pau* / extent of clear zone between *Pau* and soil isolate/ depth of lawn growth: -, none; +, 1-2 mm; ++ 2-5 mm, +++ 5-10 mm, and ++++, >10-20 mm and +++++, > 20 mm (Control NA plate with *Pau* displayed *Pau* spread of 18-20 mm from the pit with an outer clear area of 18-20 mm diameter)
